# The Physician’s Pocket Day-book

**Published:** 1888-12

**Authors:** 


THE PHYSICIAN'S POCKET DAY-BOOK, designed by C. Henri Leonard, M. A., M. D. Size, 7½ inches long, 3½ inches wide and ⅜ of an inch thick. Bound in red Morocco for the pocket, pencil loop and flap, red edges. Price $1.00 postpaid. The Illustrated Medical Journal Company, Publishers, Detroit. 1888.

This is the tenth year of issue of this popular day-book, which contains several new features. Besides accommodating daily charges for thirteen months for fifty families, and the other usual memorandum pages, it has a very complete list of doses of old and new drugs: poisons and their antidotes, tried tests for urinary deposits, chemical and microscopical, obstetric calendar, disinfectants for the sick room and vaults, tables of weights and measures, table of eruptive fevers and drops in a drachm of fluid medicines.

